# Validation of a smartphone-based EEG among people with epilepsy: A prospective study

**DOI:** 10.1038/srep45567

**Published:** 2017-04-03

**Authors:** Erica D. McKenzie, Andrew S. P. Lim, Edward C. W. Leung, Andrew J. Cole, Alice D. Lam, Ani Eloyan, Damber K. Nirola, Lhab Tshering, Ronald Thibert, Rodrigo Zepeda Garcia, Esther Bui, Sonam Deki, Liesly Lee, Sarah J. Clark, Joseph M. Cohen, Jo Mantia, Kate T. Brizzi, Tali R. Sorets, Sarah Wahlster, Mia Borzello, Arkadiusz Stopczynski, Sydney S. Cash, Farrah J. Mateen

**Affiliations:** 1 Department of Neurology, Massachusetts General Hospital, Boston, MA, USA; 2 Division of Neurology, Sunnybrook Health Sciences Centre, University of Toronto, Toronto, ON, Canada; 3 Department of Pediatrics and Child Health, University of Manitoba, Winnipeg, MB, Canada; 4 Department of Biostatistics, School of Public Health, Brown University, Providence, RI, USA; 5 Department of Psychiatry, Jigme Dorji Wangchuck National Referral Hospital, Thimphu, Bhutan; 6 Division of Neurology, Toronto Western Hospital, University of Toronto, Toronto, ON, Canada; 7 Department of Neurology, University of Washington, Seattle, WA, USA; 8 Department of Applied Mathematics and Computer Science, Technical University of Denmark, Kgs. Lyngby, Denmark

## Abstract

Our objective was to assess the ability of a smartphone-based electroencephalography (EEG) application, the Smartphone Brain Scanner-2 (SBS2), to detect epileptiform abnormalities compared to standard clinical EEG. The SBS2 system consists of an Android tablet wirelessly connected to a 14-electrode EasyCap headset (cost ~ 300 USD). SBS2 and standard EEG were performed in people with suspected epilepsy in Bhutan (2014–2015), and recordings were interpreted by neurologists. Among 205 participants (54% female, median age 24 years), epileptiform discharges were detected on 14% of SBS2 and 25% of standard EEGs. The SBS2 had 39.2% sensitivity (95% confidence interval (CI) 25.8%, 53.9%) and 94.8% specificity (95% CI 90.0%, 97.7%) for epileptiform discharges with positive and negative predictive values of 0.71 (95% CI 0.51, 0.87) and 0.82 (95% CI 0.76, 0.89) respectively. 31% of focal and 82% of generalized abnormalities were identified on SBS2 recordings. Cohen’s kappa (κ) for the SBS2 EEG and standard EEG for the epileptiform versus non-epileptiform outcome was κ = 0.40 (95% CI 0.25, 0.55). No safety or tolerability concerns were reported. Despite limitations in sensitivity, the SBS2 may become a viable supportive test for the capture of epileptiform abnormalities, and extend EEG access to new, especially resource-limited, populations at a reduced cost.

More than 80% of the 50 million people with epilepsy (PWE) worldwide live in low- and middle-income countries (LMICs)^1^. In high-income countries, electroencephalography (EEG) recordings are a standard of care for the diagnosis of seizures, aiding in the diagnosis of epilepsy, the classification of seizures and spell types, and the selection of appropriate antiepileptic drugs (AEDs)^2^,^3^,^4^,^5^. A recent survey^6^ of key respondents from 37 countries found that availability of EEG is 63% in low-income and 71% in lower middle-income countries. Waiting times are particularly long in the public sector, and only 40% of physicians from low-income countries perceive EEG as affordable^6^. Barriers to EEG services include problems that are difficult for the individual patient or clinician to fix: inconsistent electricity supplies, the high cost of EEG equipment, the low numbers of skilled technologists and interpreters of EEG recordings, and the centralization of services in cities.

The computational power available in consumer-grade mobile phones and tablets has increased dramatically in recent years. By 2021, there will be an estimated 6.1 billion smartphone owners worldwide^7^. For many people in LMICs, smartphones are the first and primary gateway to the internet, leapfrogging desktop and laptop computers. Personal mobile devices may now be used as a low-cost data capture, processing, and transmission platform for EEG. Similarly, the development of mobile-compatible electrode caps offers an opportunity for complete EEG systems that are low-cost, portable, and user-friendly in comparison to standard EEG technology.

Smartphone-based EEG could increase the availability of EEG services in remote populations of LMICs. Here, we assess the capacity of a new software application using a 14-lead headset connected wirelessly to an Android device, comparing the detection of electrophysiological abnormalities as recorded by the smartphone-based versus standard EEG among PWE in a lower middle-income country.

## Methods

### Study Site and Participant Enrolment

We recruited participants in the Himalayan Kingdom of Bhutan (total population 764,000)^8^. Bhutan was selected as a representative lower middle income country with a predominantly rural and remote population, dearth of practicing neurologists, lack of access to routine standard EEG, and endemic neurocysticercosis as a cause of epilepsy^9^. Participant enrolment and data collection took place at the Jigme Dorji Wangchuck National Referral Hospital in Thimphu, the capital city (July 2014-April 2015). PWE or suspected seizures of any age were recruited based on physician, health care worker referral from the National Referral Hospital Departments of Psychiatry and Pediatrics, the Institute of Traditional Medicine Services, and an existing epilepsy patient registry in Bhutan. Referrals also came through the “word of mouth” including advertisement through the local English language newspaper and Bhutanese radio stations. Participants were reimbursed the equivalent of nine United States dollars (USD) for their travel expenses.

### Equipment

The Smartphone Brain Scanner-2 (SBS2) is a software and hardware application for EEG that operates on mobile devices^10^. The software is available under Massachusetts Institute of Technology License and the hardware platform is available under CERN Open Hardware License (https://github.com/SmartphoneBrainScanner). The software framework supports data processing tasks such as data acquisition, filtering, recording, and real-time artifact removal. The code is written in QT C++, runs on desktop Operating Systems, including Windows, OSX, Linux as well as the most popular mobile operating system, Android. The software can be extended to work with different hardware configurations. In the present study, combined EPOC+ neuroheadset (www.emotiv.com, Emotiv Systems, Australia) and EasyCap recording cap (http://easycap.brainproducts.com, EasyCap, Germany) hardware was used.

**Figure 1:**
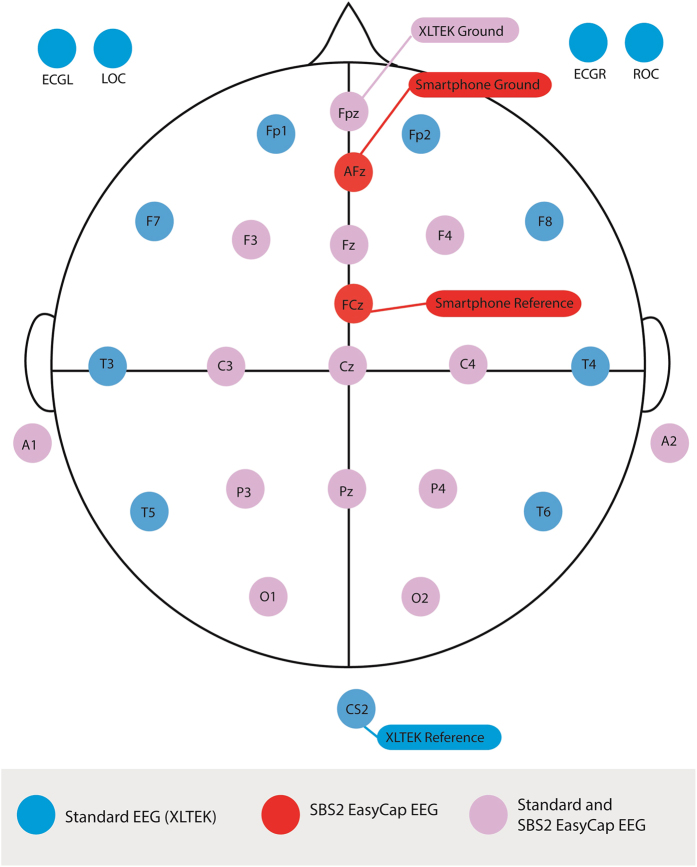
Schematic of electrode placement for SBS2 and standard EEG. ECGL: left electrocardiogram lead, ECGR: right electrocardiogram lead, ROC: right electrooculogram lead, LOC: left electrooculogram lead.

An Android tablet (Nexus 7 2013 model, Google, California) was used for data collection. Head circumference-matched EasyCap headsets with ring electrodes aligned to the International 10–20 system and positioned at F3, C3, P3, O1, F4, C4, P4, O2, Fz, Cz, Pz, Fpz, A1, and A2 (Fig. 1) were used. FCz and AFz served as the reference and ground electrodes, respectively. Five sizes of EasyCap headsets were available (range 40 cm to 56 cm) with head circumferences matched to ±2 cm to the cap of choice. These caps are “off-the-shelf” models, originally configured for videogaming rather than medical use. Impedances of all electrodes were below 5 kΩ at the start of each recording. Raw EEG data were obtained with a sampling rate of 128 Hz, and were wirelessly transmitted to a receiver module connected to the Android tablet.

A stationary Xltek EEG system (Xltek, Natus Incorporated, California) was used to record standard EEGs, using 21 scalp leads placed according to the 10–20 system, as well as bilateral electrooculogram and electrocardiogram leads.

### Data Acquisition

Each participant completed a structured questionnaire for clinician-investigators to obtain the clinical epilepsy history, and underwent consecutive SBS2 EEG and standard EEG recording. Recordings were completed sequentially at the same appointment with minimal interruption between tests whenever possible in the same study area. The order of testing was convenience-based. Each participant was supine on a hospital bed, and given instructions to minimize movements and close his or her eyes for the duration of the recording. Each recording captured wakefulness and, when possible, sleep, and was planned to last a minimum of 20 minutes and not longer than 30 minutes. Recordings were completed during the daytime in a dedicated study room in the Department of Psychiatry at the National Referral Hospital.

Staff without formal EEG training, including Bhutanese research assistants as well as medical students and neurology residents, performed SBS2 EEG recordings. Training in SBS2 EEG was less than one hour in duration and involved observing smartphone EEG being administered and/or watching an instructional video online. Standard EEG recordings were completed on Xltek machines by board-certified EEG technologists or supervised research staff and carried out in accordance with the standards of the American Clinical Neurophysiology Society^11^.

EEG files were coded by participant number and stored on encrypted, password-protected computers and external hard drives. Files were securely transferred to readers using the web-based file sharing application, Syncplicity (https://my.syncplicity.com)

SBS2 data were analyzed offline using open-source EDFbrowser software (Teunis van Beelen, available at http://www.teuniz.net/edfbrowser/) (ECWL), Profusion 3.0 software from Compumedics (Compumedics, Abbotsford, Australia) (ASPL), and Persyst software (Persyst, Prescott, USA) (ADL, RZ). The software used for SBS2 data interpretation was based on neurologist preference. Standard EEG data were analyzed offline using Natus Neuroworks software (Natus Medical Incorporated, Pleasanton, USA).

### EEG Interpretation

Board-certified pediatric (ECWL, RT) and adult neurologists (AJC, SSC, ASPL, ADL, LL, RZ, EB) interpreted the SBS2 and standard EEG recordings. EEGs were distributed to readers in no particular order. Readers were masked to clinical data other than age and comments added to standard recordings by the registered EEG technologists (JMC, JM). Readers were instructed to categorize the EEGs and record their interpretation on a standardized spreadsheet. Recordings were classified as normal or abnormal overall, and abnormalities were classified as epileptiform and/or non-epileptiform. Readers were able to enter notes clarifying their interpretation. Each SBS2 recording was read once. Each standard EEG was independently assessed by ≥2 neurologists. A third neurologist or a group of neurologists resolved discrepancies in standard EEG interpretation. The individual interpreting a given participant’s SBS2 EEG was blinded to the findings from their standard EEG and vice versa. Due to the different electrode arrays, EEG interpreters could not be blinded to the type of study being performed. All EEG interpretation occurred on desktop computers, and viewing montages were selected at the discretion of the interpreting neurologist.

Participants were excluded from the analysis if: (1) they did not undergo both standard and smartphone EEG, or if either recording was unavailable (n = 52), or (2) the smartphone EEG recording was <50% of the targeted recording time of 20 minutes (n = 11).

### Patient Follow up

Participants were provided the results of their standard EEG as well as the results of ancillary tests performed as part of the broader study, including brain MRI and blood tests for *Taenia solium*, at follow-up visits. Neurologists made treatment recommendations as necessary based on clinical judgment and test findings.

### Data Analysis

The final interpretation of the standard EEG was used as the gold standard in the assessment of the SBS2 EEG. The sensitivity, specificity, and positive and negative predictive values for the SBS2 EEG versus the standard EEG were calculated for the detection of all types of electrophysiological abnormalities, epileptiform abnormalities, and non-epileptiform abnormalities. Inter-rater reliability measured by Cohen’s kappa (κ) was used to compare agreement between readings of two independent readers of standard EEG and across EEG types. Cohen’s kappa takes into account the possible randomly occurring agreement between readers, hence providing a better measure of agreement than a simple percentage. *Post ho*c analyses included a comparison of focal versus generalized discharge capture and a description of the SBS2 EEGs excluded for limited duration of recording time. A p-value of <0.05 with two-tailed probability was considered statistically significant. All analyses were performed using the programming language R (Vienna, Austria).

### Standard Protocol Approvals, Registrations, and Patient Consents

The Research Ethics Board of the Ministry of Health of Bhutan, the Partners Healthcare Institutional Review Board of the Massachusetts General Hospital, USA, and the Research Ethics Board of the University of Ottawa, Canada approved the study. All research activities were performed in accordance with relevant guidelines and regulations outlined in the approved protocol. All participants provided informed consent or, when appropriate, assent with proxy consent from a family member.

## Results

**Figure 2:**
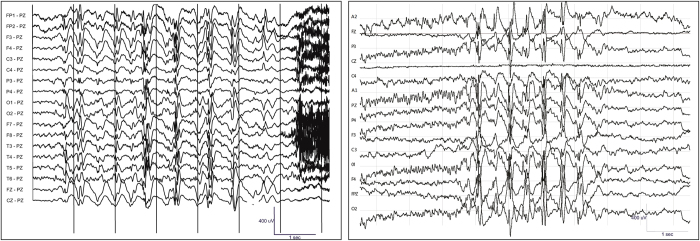
Frontal spike and wave discharges captured by (**A**) standard and (**B**) SBS2 EEG recordings from a Bhutanese participant during two separate recordings. The reference electrode is FCz.

**Figure 3:**
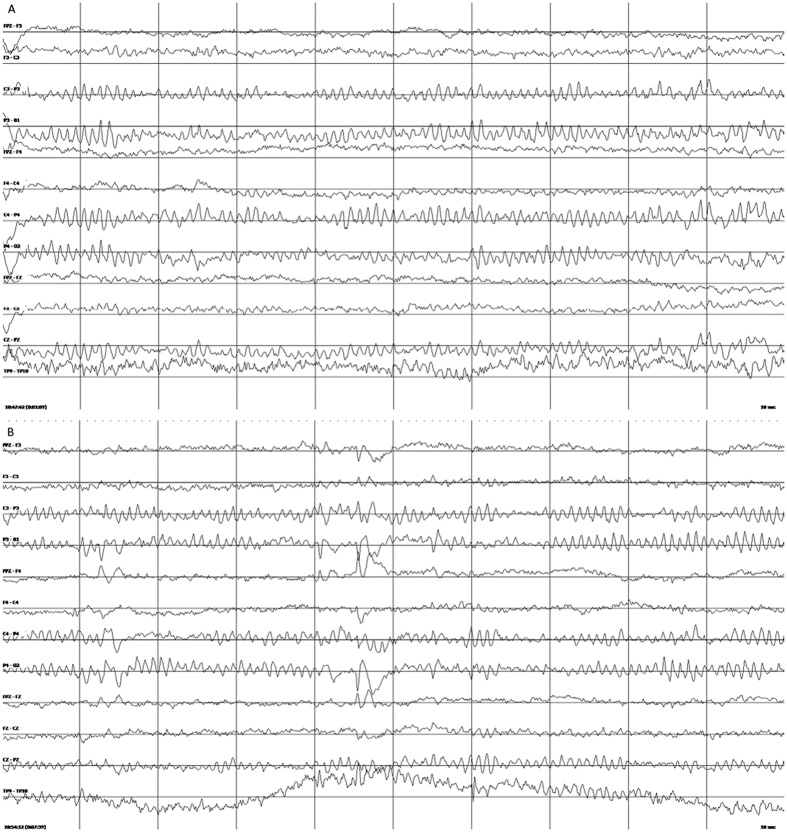
SBS2 EEG interpreted as (**A**) normal 9 Hz posterior dominant rhythm, and (**B**) a left frontal (F3) sharp wave in Bhutanese participant.

205 participants (54% female, median age 24 years) completed both SBS2 and standard EEG (Table 1). There were no safety or tolerability concerns reported. One participant had a tonic seizure during standard EEG recording, while all other recordings were interictal. The mean length of SBS2 EEG recording was 21.3 minutes (median 21.0 minutes, standard deviation 3.2 minutes), and the mean length of the standard EEG recording was 26.0 minutes (median 25.2 minutes, standard deviation 8.1 minutes). A comparison between the outputs of a standard versus SBS2 EEG is provided in Fig. 2. Representative EEG tracings from the SBS2 are provided in Fig. 3.

### EEG Interpretations

Epileptiform discharges were present on 14% and 25% of SBS2 and standard EEGs, respectively. The SBS2 had 39.2% sensitivity (95% confidence interval (CI) 25.8%, 53.9%) and 94.8% specificity (95% CI 90.0%, 97.7%) for the detection of epileptiform discharges. The overall percent agreement for the detection of epileptiform abnormalities was 81% (166/205), the positive percent agreement was 39% (20/51) and the negative percent agreement was 95% (136/154). Contingency tables for the detection of all abnormalities, epileptiform abnormalities and non-epileptiform abnormalities by the standard and SBS2 EEGs are presented in Table 2. Sensitivity, specificity, and positive and negative predictive values are reported in Table 3. Cohen’s kappa (κ) for the SBS2 EEG interpretation and standard EEG final interpretation for the abnormal versus normal outcome was κ = 0.39 (95% CI 0.26, 0.52). Cohen’s kappa (κ) for the agreement between the SBS2 EEG and standard EEG interpretations for the epileptiform versus non-epileptiform outcome was κ = 0.40 (95% CI 0.25, 0.55).

In *post hoc* analyses, we assessed whether epileptiform abnormalities captured were focal or generalized. Among the 51 standard EEGs with epileptiform abnormalities, 42 had focal and 9 had generalized abnormalities. On the SBS2 recordings, 31% (13/42) of focal and 82% (7/9) of generalized were identified. When only pediatric participants (n = 49) were considered, the sensitivity for the detection of epileptiform abnormalities by the SBS2 EEG was 0.56 (95% CI 0.31, 0.79) and the specificity was 0.87 (95% CI 0.61, 0.92).

### Agreement among EEG readers

The interpretation of the SBS2 EEGs was divided among four neurologists, who each read 7–59% of the recordings. The standard EEG interpretations were divided among nine neurologists, who each read 1–26% of the recordings. All standard EEGs were interpreted by two separate neurologists. Cohen’s kappa (κ) for the two independent interpretations classifying each standard EEG as normal or abnormal was 0.60 (95% CI 0.49, 0.71). Cohen’s kappa (κ) for the two independent interpretations classifying each standard EEG as epileptiform or non-epileptiform was 0.60 (95% CI 0.47, 0.72).

## Discussion

In this clinical validation study of the SBS2, we found a low to moderate sensitivity but high specificity for the detection of epileptiform abnormalities, compared to standard EEG, in our study population of PWE. For the combined endpoint of epileptiform and non-epileptiform abnormalities, the SBS2 had low sensitivity but high specificity for detection. Generalized epileptiform abnormalities were more often captured than focal abnormalities on SBS2 when compared to standard EEG. Our results support the SBS2 as a pragmatic option for some PWE in LMICs to receive a confirmation of clinically suspected epilepsy; however, the SBS2 is not presently an ideal screening test for epilepsy. With modifications, the SBS2 may be particularly useful in both high and low-income settings where standard EEG is unavailable, and may have applications for home-based assessment for suspected seizure disorders.

Strengths of this study include a relatively large sample size, the inclusion of a community-dwelling sample, the inclusion of all age groups, and the contributions of a large team of academic neurologist EEG readers. The pragmatic design of the study demonstrated the feasibility of acquiring SBS2 EEG using a portable tablet and EasyCap system in a LMIC population. The detection of epileptiform discharges is a practical and clinically relevant endpoint that is valuable to the diagnosis of PWE.

However, there are several limitations to the study design as well. These include the sequential rather than simultaneous recording of EEGs and the assessment of the complete SBS2 system, including software and hardware components as a whole. Given the sequential nature of recordings, intermittent events occurring during the first recording may not be captured on the second and vice versa. This makes direct comparison of sequential at times imperfect. Both the duration of the abnormal discharges and the duration of the recordings are additional variables that may have impacted our results beyond the device characteristics themselves. Our study therefore also highlights the technical challenges of new mobile device testing in the environments where they could ultimately be most beneficial clinically.

We used Xltek, an EEG system similar to one that a patient would encounter at a high-income country’s medical center with conventional EEG services as our “gold standard.” Alternatives, including dense array EEG and intracranial electrode monitoring may be optimal gold standard devices in other situations, but are not the standard of care for clinical practice. The interpretation of standard, clinical EEG is also very subjective, and the inter-rater reliability of experienced neurologists interpreting EEG is often poor^12^,^13^. In one single-center study, in which six board-certified neurophysiologists classified 300 EEGs into seven diagnostic categories, the aggregated Fleiss kappa for interrater agreement was 0.44^12^. Our study had higher interrater agreement for standard EEG. This is likely due to the fewer categories of classification (normal, epileptiform abnormalities, or non-epileptiform abnormalities) in our analyses. One proposed solution is automated interpretation of EEG recordings. Although automated detection of discharges and non-reader dependent quantitative assessment of EEG output is an area of ongoing investigation^14^,^15^, it is not yet accepted for clinical assessment of PWE.

We believe the largest limitation to the use of SBS2 as an alternative to standard EEG equipment is the low to moderate sensitivity of the SBS2 for the capture of EEG abnormalities. Technical limitations, including the 14-lead headset without temporal or complete frontopolar coverage and the inability to monitor headset connectivity in real-time may have contributed to the reduced sensitivity of the SBS2. Some SBS2 recordings were of shorter duration than targeted, and those less than <50% of the targeted 20 minute duration were removed from analysis. *Post hoc* analyses found that inclusion of these shorter recordings modestly reduced sensitivity and most of the short recordings were interpreted as normal. Other possibilities for reduced sensitivity include that the software may have less capability to process cortical signals and that neurologists interpreting the SBS2 recordings were less likely to report an epileptiform discharge on an SBS2 compared to a more conventional system. EEGs were distributed based on convenience, but given the relatively small number of SBS2 readers, we cannot rule out reader-dependent differences in the interpretation of the EEGs.

The SBS2 provides distinct advantages, several with particular importance to resource-limited settings. The most notable advantage of the SBS2 is cost savings compared to standard EEG. The SBS2 equipment costs approximately 300 USD per device, making the technology more than twenty-fold less costly than standard EEG equipment. Since medical equipment for the care of patients with brain disorders is often not prioritized in LMICs^16^,^17^, low-cost technologies at the “point of care” hold great potential for uptake^18^. More than 95% of physicians in training to become neurologists in sub-Saharan Africa in 2015 reported owning a personal smartphone^19^. Moreover, the potential to record without electricity for up to twelve hours (i.e. the battery life of the phone or tablet) allows the SBS2 EEG to be used facilely in remote settings since internet connectivity is required only to transfer files. Operators with only basic training can use the SBS2, as in this study, making the device’s use by community healthcare workers possible. Future uptake of such a device could thus be rapid if distribution is properly designed.

Several mobile EEG systems are currently available or in the pipeline, including Mobita (Twente Medical Systems international), TREA (Natus Neurology), Trackit (Lifelines Neurodiagnostic Systems Inc.) and Safiro (Compumedics). An important advantage of the SBS2 EEG is that the open-source software can be downloaded to any Android device; however, the EasyCap and Emotiv components would still need to be purchased. By comparison, most market EEG systems use proprietary software for data capture and viewing. Our study compares a mobile phone-based EEG system to a standard EEG system in a lower income country among people with epilepsy who have not had prior access to EEG. Nonetheless, each of these mobile systems is ripe for future iterative development for PWE, as limitations in implementation and performance are carefully addressed in clinically relevant studies.

As is well-recognized, the feasibility of standard EEG in LMICs and remote settings is limited by cost, reliance on unpredictable electricity, increased preparation time for recording, the production of larger data files produced that cannot be transferred via email, and lack of technical support in the case of equipment failure. These examples have been presented from around the world but may be most relevant to populations in LMICs. In Nigeria, a retrospective study^20^ found that EEG resources were under-utilized by 75% due to a lack of recording paper, electrode gel, increasing cost of EEG, and strikes by hospital staff. A case series from a tertiary hospital in Harare, Zimbabwe found that only 4.2% of EEG referrals were from district hospitals, finding that rural dwellers were least able to access EEG services when they existed only in the capital city^21^. Among five southern Caribbean countries, EEG services were available in only two, and the percentage of patients receiving EEG who required one ranged from 10–68% across the five countries^22^.

Future directions for research on the SBS2 EEG include the assessment of new headset devices with customized lead configurations, the assessment of new mobile computing devices including iOS™based operating systems, user-friendly updates to the application, and the development of automated and “crowd-sourced” EEG interpretation. Further investigation is needed to identify what populations of PWE might benefit most from access to SBS2, and which epilepsy syndromes and categories are most amenable to evaluation by the SBS2. Based on our findings, we believe a generalized epilepsy syndrome such as absence seizures in childhood (i.e. so-called staring spells) may be an obvious patient group of benefit given the usually high frequency of events, the generalized epileptiform pattern, and the ability to intervene early to prevent school dropouts and delays in learning.

Advances in technology must parallel the development of a healthcare workforce skilled in the implementation and assessment of new health technologies, including transferrable technologies with health relevance. On the horizon of bringing EEG acquisition to the smartphone of any interested person, we provide a “proof of concept” that smartphone-based EEG systems are feasible and may soon allow PWE worldwide access to EEG, including in locations where such services have not previously existed.

## Additional Information

**How to cite this article:** McKenzie, E. D. *et al*. Validation of a smartphone-based EEG among people with epilepsy: A prospective study. *Sci. Rep.* **7**, 45567; doi: 10.1038/srep45567 (2017).

**Publisher's note:** Springer Nature remains neutral with regard to jurisdictional claims in published maps and institutional affiliations.

## Figures and Tables

**Table 1:** Baseline characteristics of participants who underwent SBS2 and standard EEG testing (n = 205).

Age, median (interquartile range) years	24 (18–32)
Female	111 (54%)
Previously diagnosed with epilepsy	188 (92%)
Most recent seizure	
Within past week	57 (28%)
Within past month	61 (30%)
Within past year	27 (13%)
More than one year ago	60 (29%)
Seizure Characterization	
Loss of consciousness	142 (69%)
Staring spells	34 (17%)
Falling with stiffening and shaking	115 (56%)
Current seizure treatment	
No AEDs	19 (9%)
Carbamazepine	69 (34%)
Lamotrigine	8 (4%)
Levetiracetam	27 (13%)
Phenobarbital	42 (20%)
Phenytoin	77 (38%)
Benzodiazepines	17 (8%)
Other AEDs†	3 (1%)

SBS2: Smartphone Brain Scanner-2.

EEG: Electroencephalogram.

AEDs: Anti-epileptic drugs.

^†^Other AEDs included topiramate (2) and vigabatrin (1).

**Table 2:** Contingency tables comparing outcome frequencies on SBS2 and standard EEG.

**Outcome: Any abnormalities (epileptiform or non-epileptiform) detected vs. normal recording**			
		Standard EEG	
		Abnormal	Normal
SBS2 EEG	Abnormal	42	20
	Normal	37	106
Outcome: epileptiform discharges vs. no epileptiform discharges detected			
		Standard EEG	
		Epileptiform	No epileptiform
SBS2 EEG	Epileptiform	20	8
	No epileptiform	31	146
Outcome: non-epileptiform abnormalities vs. no non-epileptiform abnormalities detected			
		Standard EEG	
		Non-epileptiform abnormalities	No non-epileptiform abnormalities
SBS2 EEG	Non-epileptiform abnormalities	20	21
	No non-epileptiform abnormalities	35	129

**Table 3:** Measurement of sensitivity and specificity of SBS2 EEG versus standard EEG for the detection of all abnormalities, epileptiform discharges and background abnormalities (n = 205).

	All abnormalities (Epileptiform or non-epileptiform)		Epileptiform discharges		Non-epileptiform abnormalities	
		95% CI		95% CI		95% CI
Sensitivity	0.53	(0.41, 0.64)	0.39	(0.26, 0.54)	0.36	(0.24, 0.50)
Specificity	0.84	(0.77, 0.90)	0.95	(0.90, 0.98)	0.87	(0.79, 0.91)
Positive predictive value	0.67	(0.55, 0.79)	0.71	(0.51, 0.87)	0.49	(0.33, 0.65)
Negative predictive value	0.74	(0.6, 0.81)	0.82	(0.76, 0.89)	0.79	(0.72, 0.85)
